# Minimum and Maximum Void Ratios of Sand–Rubber and Crushed Concrete–Rubber Mixtures

**DOI:** 10.3390/ma19091721

**Published:** 2026-04-23

**Authors:** Magdalena Kowalska, Bartosz Bdzionek, Katarzyna Gabryś, Iwo Zatorski, Cristiana Ferreira

**Affiliations:** 1 Department of Geotechnics and Roads, Faculty of Civil Engineering, Silesian University of Technology, Akademicka 5, 44-100 Gliwice, Poland; 2 Faculty of Civil Engineering, Silesian University of Technology, Akademicka 5, 44-100 Gliwice, Poland; 3 Department of Geotechnics, Institute of Civil Engineering, Warsaw University of Life Sciences, Nowoursynowska 159, 02-776 Warsaw, Poland; katarzyna_gabrys@sggw.edu.pl; 4 Institute of Civil Engineering, Warsaw University of Life Sciences, Nowoursynowska 159, 02-776 Warsaw, Poland; s210358@sggw.edu.pl; 5 CONSTRUCT-GEO, Faculty of Engineering, University of Porto (FEUP), Rua Roberto Frias, s/n, 4200-465 Porto, Portugal; cristiana@fe.up.pt

**Keywords:** maximum and minimum void ratios, scrap tyre rubber, recycled concrete aggregate, Proctor test, vibration

## Abstract

**Highlights:**

**What are the main findings?**

- Boundary void ratios of well-graded RCA, TDA and RCA-TDA were determined using various methods.
- The lowest *e_min_* was achieved in Proctor compaction for RCA and RCA-TDA and in static loading for TDA.
- Dry RCA, TDA, and RCA-TDA reach *e_max_* by cylinder inversion, though particle segregation is induced.

**What are the implications of the main findings?**

- Particle characteristics and water content significantly influence *e_min_* and *e_max_* of recycled geomaterials.
- Standard vibration overestimates *e_min_* for RCA, TDA and RCA-TDA but is effective in sand–TDA.
- The minimum void ratio concept for rigid soils is not directly applicable to rubber-rich geomaterials.

**Abstract:**

There are no unique and universally accepted procedures for the determination of the maximum and minimum void ratios, *e_max_* and *e_min_*. This issue is particularly pertinent in the characterisation of the alternative sustainable materials examined in this study: well-graded tyre-derived aggregate (TDA), recycled concrete aggregate (RCA) and their mixtures (RCA-TDA), with a rubber content by weight of *Χ_M_* = 11, 23 and 55%. Uniformly graded TDA–sand mixtures with *Χ_M_* = 0, 15, 27, 42, and 100% were also considered. The results from dry and moist samples were compared with void ratios obtained after Proctor compaction and static loading. It was found that, in contrast to vibration for sand and sand–TDA mixtures, the most efficient densification techniques involve impact compaction at the optimum water content for RCA and RCA-TDA and static loading for TDA. Inversion of dry RCA, TDA and RCA-TDA samples in a graduated cylinder was the most effective to consistently achieve *e_max_* but induced visible segregation. Unlike sand–rubber mixtures, well-graded RCA-TDA did not exhibit a threshold rubber content at which *e_max_* and *e_min_* fell below those of RCA and TDA alone, suggesting reduced segregation. The findings offer practical guidance for improving specimen preparation reproducibility in the laboratory.

## 1. Introduction

The void ratio is a fundamental physical parameter of granular soils. In a non-cohesive, permeable soil with solid, incompressible, and non-deformable grains, it is assumed to be bounded by the minimum and maximum values, *e_min_* and *e_max_*, respectively. In practice, these boundary values are required to assess the relative density, *I_D_* or *D_r_*, of natural soil deposits or man-made fills and to estimate their densification potential under static or dynamic loading. In laboratory practice, *e_min_* and *e_max_* are further necessary to prepare comparative specimens at a target *I_D_* value.

The minimum and maximum void ratios of a given soil can be determined based on its specific density *ρ_s_* [g/cm^3^], water content *w* [%], maximum and minimum bulk densities, *ρ_max_* [g/cm^3^] and *ρ_min_* [g/cm^3^], or maximum and minimum dry densities, *ρ_d,max_* [g/cm^3^] and *ρ_d,min_* [g/cm^3^], respectively:

(1) $$e_{min}=\frac{\varrho_{s}\cdot \left(100+w\right)}{100\cdot \varrho_{max}}-1=\frac{\varrho_{s}}{\varrho_{d,max}}-1,$$

(2) $$e_{max}=\frac{\varrho_{s}\cdot \left(100+w\right)}{100\cdot \varrho_{min}}-1=\frac{\varrho_{s}}{\varrho_{d,min}}-1.$$

In the laboratory, various methods can be used to achieve the loosest and densest grain arrangements. Some of these methods can be found in national standards, as described in Section 2.2. Depending on the country, the standard test methods differ significantly in terms of equipment, test procedures, and applicability criteria for specific soil types, thereby directly influencing the results. As there are no unique methods for determining *ρ_min_* and *ρ_max_*, researchers can choose or adapt procedures suited to the material under investigation. On the other hand, the lack of precise and consistent instructions hinders comparing and contrasting results across publications. This limitation is further compounded when atypical, non-textbook geomaterials are analysed, such as recycled aggregates, which fall outside the scope of conventional standards.

In this study, the focus is on two alternative materials derived from waste: rubber produced from end-of-life car tyres (also known as ‘tyre-derived aggregate’, TDA) and recycled concrete aggregate (RCA). Both can be used as sustainable substitutes for natural soils (sands and gravels), especially in applications involving cyclic and dynamic loading, such as geotechnical seismic isolation systems [1,2,3,4,5,6].

Tyre-derived aggregate, TDA, consists primarily of rubber with varying proportions of textile and steel cord. According to the European standard EN 14243 [7] and based on the size of the particles, TDAs can be classified as cuts (≥300 mm), shreds (20–400 mm), chips (10–50 mm), granulates (0.8–20 mm) or powder (<0.8 mm). Most geotechnical applications and large-scale physical tests refer to shreds and chips, given their lower manufacturing energy requirements and cost-effectiveness. However, significant steel and textile cord content in their composition makes the material highly heterogeneous and raises concerns regarding the negative environmental impact of metal leaching (iron and manganese) in applications below the groundwater table [8,9,10]. Steel cord is also believed to be responsible for the self-ignition incidents reported in 1995, when the first TDA road embankments were built in the USA [11,12]. Consequently, granulates and powders made almost entirely of pure rubber have recently attracted increasing attention. These fractions are readily available from tyre recycling companies in various fractions and are particularly suited to small-scale laboratory testing, where the maximum particle size is constrained by equipment dimensions. TDAs differ significantly from natural mineral soils in terms of their lower specific gravity [13], lower thermal conductivity [14], lower initial stiffness moduli and slower stiffness degradation with shear strain [15,16,17,18,19,20], and larger damping ratios [15,17]. TDA is also susceptible to significant creep deformation [21]. Although, like other granular soils, rubber particles can be assumed to be incompressible under typical engineering loading conditions, they are viscoelastic and deformable, capable of changing shape without fracture. This feature strongly influences the boundary void ratio values investigated in the present study.

In some practical applications, the low stiffness of TDA represents a significant limitation. This problem can be mitigated by mixing TDA with coarse mineral soil, for which natural sand or gravel are conventionally used. However, as mining of these aggregates is both expensive and environmentally detrimental [22,23,24], it is urgent to find alternative solutions. One of them involves replacing natural aggregates with crushed recycled concrete. Compared to natural sand and gravel, the properties of RCA are inherently more variable, depending on several factors, such as the origin of the parent materials, the presence of impurities, or the production method [25,26]. RCA particles are typically angular with rough surface texture, often retaining residual mortar on their surfaces [27]. The studies of de Juan and Gutiérrez (2009) [28] and Mazhoud et al. (2022) [29] showed higher water absorption and porosity and lower particle density of RCA compared to natural aggregates.

The existing literature has focused predominantly on the reuse of RCA in concrete [30,31,32,33,34,35,36]. These studies indicated a rather negative impact on both the mechanical performance and workability of concrete, which can be reduced by applying modern pre-treatment techniques and appropriate on-site handling [30]. In contrast, the behaviour of RCA as an unbound geomaterial has received comparatively limited attention [37,38,39]. Existing studies show that the California Bearing Ratio and resilient modulus of RCA can successfully meet the typical requirements set by road authorities [38]. Its high shear strength (angle of internal friction between 37° and 40°) and lower unit weight (compared to natural aggregates) make it an effective backfill material, with the additional benefit of reduced lateral earth pressure. RCA mixtures often require less compaction energy than natural aggregates to reach maximum dry density, which has the potential to lower construction costs. Blending RCA with softer soils or other recycled materials, such as crushed glass, can significantly enhance the shear resistance of binary mixtures. The challenges associated with the residual mortar, such as lower particle strength and reduced interparticle contact stiffness compared to quartz sand [37], or possible clogging with fines when used in drainage systems [39], remain valid in geotechnical applications. Nevertheless, there are significant environmental, practical, and economic advantages in using RCA that justify its further investigation as a geomaterial.

From an environmental perspective, RCA helps divert construction and demolition waste from landfills, minimises the consumption of natural resources and lowers energy demand associated with the extraction and processing of natural soils and aggregates [40]. Furthermore, RCA contributes to mitigating the construction industry’s carbon footprint, aligning with global efforts towards sustainable development and environmental stewardship [41]. Economically, RCA is often a cost-effective alternative to natural aggregates, primarily due to lower sourcing, processing, and transportation costs, particularly when produced locally [42]. The material can be processed into a wide range of particle sizes, from fine dust to larger chunks, making it suitable for diverse applications, such as road bases and subbases, building foundations and structural fill, pipe bedding and backfilling trenches, landscaping (e.g., pathways, retaining walls), and drainage systems [26].

Regarding the minimum and maximum void ratios, there are very few studies reporting these values for TDAs [43,44,45,46,47,48,49]. More common are the results for mixtures of rubber with sand or gravel [43,44,45,47,48,49,50]. The boundary void ratios are usually listed alongside other physical parameters, often without specifying the procedures used for their determination. To the best of the authors’ knowledge, published results are limited to uniformly graded rubber granulates with median particle sizes *d*_50_ between 1.5 and 14.5 mm and narrow ranges of the coefficient of uniformity *C_U_* and coefficient of curvature *C_C_* of 1.0–2.7 and 1.0–1.5, respectively. Well-graded specimens, which, according to ISO 14688 [51], should be characterised by parameters *C_U_* > 15 and *C_C_* = 1–3, have not yet been analysed.

Data on the geotechnical properties of RCA and RCA-TDA mixtures (subsequently denoted as ‘RCA-TDA’) are similarly very scarce [52,53,54], and no systematic determination of *e_min_* and *e_max_* for RCA-TDA has been reported to date. The existing research generally reports only the current void ratio and/or overall compressibility behaviour rather than the boundary values. The only exception is the prior research by one of the authors of this study [55], which reported *e_min_* and *e_max_* values for very fine RCA (*d*_50_ = 0.12–0.2 mm; *C_U_* = 2.9– 5.6; *C_C_* = 0.9–1.3) and its mixtures with up to 20% of rubber powder/granulate (*d*_50_ = 0.65–1 mm; *C_U_* = 2.4; *C_C_* = 0.7–1.2) by weight.

In the literature, no systematic study has yet examined the boundary void ratio values of TDA, RCA, or RCA-TDA with respect to the applicability of the standard procedures typically used for natural soils. The present study addresses this knowledge gap with a focus on well-graded materials. The results are compared with those for uniformly graded RCA and TDA as well as with the data for uniformly graded medium sand and sand–TDA mixtures previously investigated by Kowalska and Vrettos [15,16]. To further explore the influence of grading characteristics, two preparation methods are employed for the well-graded RCA-TDA: (i) M1—combining well-graded RCA and well-graded TDA sharing the same grain size distribution, and (ii) M2—substituting selected particle size fractions of well-graded RCA with TDA. This dual-method approach represents an additional novel contribution of the study. To provide further insight into the material behaviour under dynamic and static loading, void ratios of selected specimens were also determined after compaction with standard Proctor energy and after static loading in a shear box. The testing procedures were designed to yield *e_min_* and *e_max_* values representative of specimens with a diameter of approximately 70 mm and a height of about 140 mm. The results obtained establish a preliminary experimental basis for future research on the mechanical properties of these sustainable materials.

It should be noted that selected results for well-graded RCA-TDA mixtures prepared using method M1 will be published in conference proceedings [56].

## 2. Materials and Methods

### 2.1. Materials

Tyre-derived aggregates (TDAs) in fractions of 0–1, 1–4, and 3–6 mm were obtained from the local company Orzeł S.A. (Poniatowa, Poland) These were produced by ambient-temperature shredding and sieving of car tyres; free steel and textile cords were removed using magnets and air classifiers, respectively. Recycled concrete aggregate (RCA) was delivered by Holcim Polska S.A. (Kielce, Poland) as a product of the demolition of a shopping centre in Poland, specifically from the concrete ceilings and columns of the above-ground car park. The material came in 3 batches of fractions of 1–30, 0.3–10, and 3–40 mm; apart from crushed concrete, these contained small amounts of other waste, such as polystyrene, hardened construction foam, paper, plastic, glass, and organic matter. The material was washed, and the impurities were manually separated and removed.

**Figure 1:**
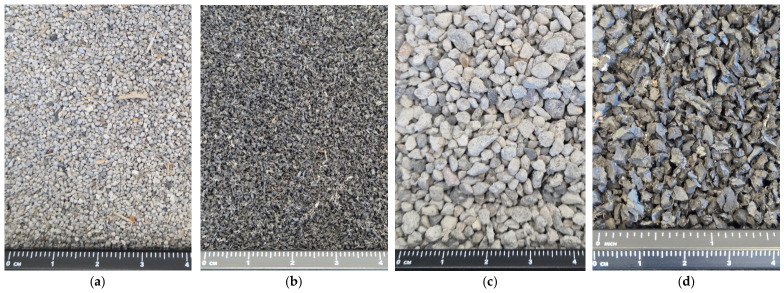
Selected fractions of the materials: (**a**) RCA 0.5–1.0 mm, (**b**) TDA 0.5–1.0 mm, (**c**) RCA 2–4 mm, (**d**) TDA 2–4 mm; photo: B. Bdzionek.

The main goal of the study was to determine the boundary void ratios necessary to prepare specimens of controlled *I_D_* for further geotechnical testing in a triaxial and a resonant column apparatus. Both pieces of equipment accommodate specimens with dimensions of 140 mm in height and 70 mm in diameter, imposing a maximum particle size limit of 11.7 mm. Taking into account the maximum available size of the rubber particles and the requirement to investigate a coarse free-draining soil for which *e_min_* and *e_max_* are applicable, only fractions between 0.063 and 6.3 mm were selected for testing. Finer particles were excluded from the study, whereas oversize concrete fragments were reduced in size using a jaw crusher. The RCA and TDA were oven-dried and separated into subfractions using sieves with openings of 0.063, 0.1, 0.25, 0.5, 1, 2, 4, 5, 5.6, and 6.3 mm. A macroscopic view of selected fractions of RCA and TDA is presented in Figure 1. It can be noticed that the RCA particles were either cubic or slightly elongated and flattened, angular, and rough. The shapes of the TDA particles were similar, but their surfaces were slightly smoother. The finer fractions of TDA contained some loose textile fibres, while in the coarser particles, the textile fibres were often embedded in the rubber mass.

**Figure 2:**
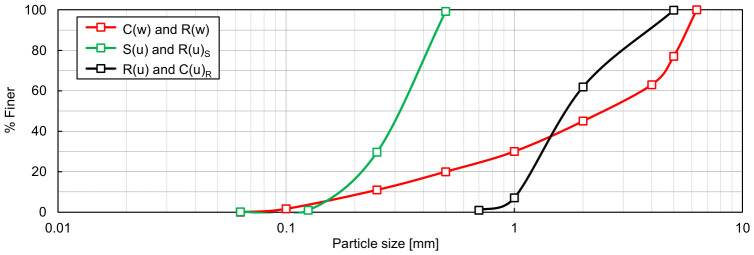
Particle size distribution of the single-material samples: C(w)—well-graded RCA, R(w)—well-graded TDA, S(u)—uniformly graded sand [15], R(u)—uniformly graded TDA [15], C(u)_R_—RCA with grading identical to R(u), R(u)_S_—TDA with grading identical to S(u).

A particle size distribution fitting the well-graded soil criteria (*C_U_* > 15 and *C_C_* = 1–3) was developed to obtain a well-graded RCA (C(w)) and a well-graded TDA (R(w)) by suitably mixing the necessary subfractions; this target distribution is represented by the red curve in Figure 2. The materials examined in the earlier works of Kowalska and Vrettos [15,16] were also adopted, namely a uniform medium fluvial sand (S(u)) and a uniform rubber granulate (R(u)). The sand, originating from Bobenheim-Roxheim in Germany, was purchased from Industrie-Sandwerke Pfalz (Bobenheim-Roxheim, Germany) (sand No. 30), while the scrap tyre rubber was supplied by the company ATB Truck S.A. (Śrem, Poland). To further elucidate how grading characteristics affect the boundary void ratios of RCA and TDA, two additional samples were prepared: RCA with the same grading curve as R(u), denoted C(u)_R_, and TDA with the grading curve as S(u), denoted R(u)_S_. The grading curves of all single-material samples considered in this study are shown in Figure 2, while their grading characteristics and specific densities are listed in Table 1. The ***ρ_s_*** values were evaluated in accordance with ISO 17892-3 [57] using a fluid pycnometer for RCA and sand and a gas pycnometer (AccuPyc II 1340, prod. Micrometrics, Norcross, GA, USA) for TDA.

Seven RCA-TDA mixtures were produced. Their particle size distributions were identical to those of samples C(w) and R(w) shown in Figure 2. Three mixtures were prepared using method M1, i.e., by combining the C(w) and R(w) samples in adequate proportions to achieve the rubber contents by weight *Χ_M_* = 11%, 23%, and 55%, where *Χ_M_* is defined according to the following formula:

(3) $${Χ}_{M}=\frac{m_{r}}{m_{r}+m_{s}}\cdot 100\%,$$

in which *m_r_* and *m_s_* are the masses of dry rubber (TDA) and soil (here: RCA) particles, respectively.

In these mixtures, every subfraction contains both RCA and TDA; consequently, the ratio of the median particle sizes of rubber *d*_50,*r*_ and soil *d*_50,*s*_, defined with the equation:

(4) $$\eta =\frac{d_{50,r}}{d_{50,s}}\;,$$

equals 1. Another three mixtures were obtained using method M2, which involved starting from sample C(w) and replacing the coarse subfractions retained on the 5.6, 5.0, or 2.0 mm sieves with the corresponding R(w) subfractions. The rubber content by weight in these mixtures was likewise 11, 23, and 55%, respectively. The last RCA-TDA mixture, again with *Χ_M_* = 11%, was also prepared using method M2, but in this case, the fine subfractions of C(w) passing the 0.25 mm sieve were substituted with the corresponding subfractions of R(w).

**Figure 3:**
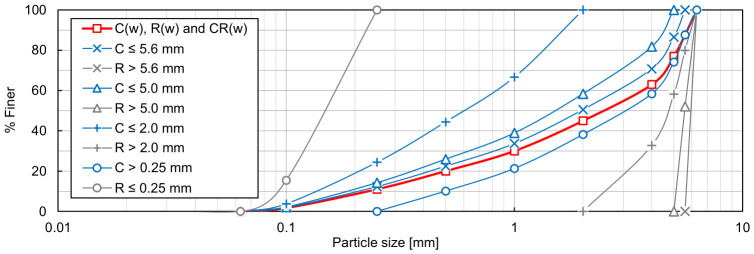
Particle size distribution of the components of CR(w) mixtures.

The particle size distributions of the RCA (C) and TDA (R) components of the RCA-TDA mixtures are presented in Figure 3, and their grading characteristics and specific densities are listed in Table 2, where the subscripts ‘s’ and ‘r’ denote the parameters of the mineral (RCA) and rubber (TDA) components, respectively. The specific densities of the mixtures, *ρ_s,mix_*, were calculated using the following equation:

(5) $$\rho_{s,mix}=\frac{1}{\frac{{Χ}_{M}}{100\cdot \rho_{s,r}}+\frac{{100-Χ}_{M}}{100\cdot \rho_{s,s}}}\;,$$

where *ρ_s,r_* and *ρ_s,s_* are the specific densities of the rubber and mineral components, respectively. The sample designations are coded in the following way: ‘CR(w)/*Χ_M_*‘ refers to mixtures prepared using method M1, while ‘CR(w)/*Χ_M_*/rubber fraction’ refers to mixtures prepared using method M2. For example, ‘CR(w)/55%’ denotes a well-graded mixture of C(w) and R(w) containing 55% of rubber by weight, whereas ‘CR(w)/23%/>5.0 mm’ denotes a well-graded mixture with 23% rubber by weight, consisting of RCA particles not exceeding 5.0 mm and TDA particles larger than 5.0 mm.

It should be emphasised that, although the RCA-TDA mixtures prepared using method M2 are well-graded, their individual RCA and TDA components cannot be classified as well-graded once the designated subfractions are removed; consequently, the *η* values of these mixtures are no longer equal to 1.

**Figure 4:**
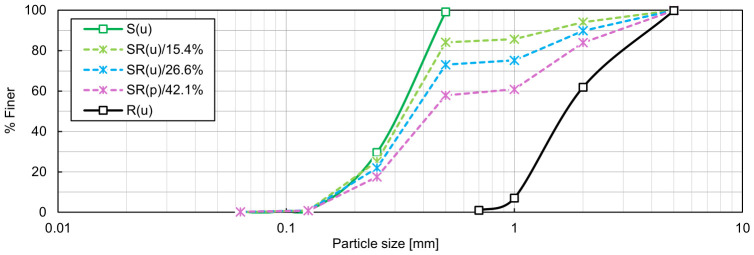
Particle size distribution of the SR mixtures.

In addition to the RCA-TDA mixtures, sand–TDA mixtures (SR(u)) were prepared by combining samples S(u) and R(u) (method M1) in suitable proportions to obtain *Χ_M_* = 15.4, 26.6, and 42.1% (the corresponding rubber contents by volume *Χ_V_* were equal to 1/3, 1/2, and 2/3, respectively), as in [15,16]. Their particle size distribution curves, grading characteristics, and specific densities are shown in Figure 4 and Table 3. Following ISO 14688-2 [51], the mixture with 42.1% rubber by weight is classified as poorly graded (p), whereas the mixtures with *Χ_M_* = 15.4% and 26.6% rubber are categorised as uniformly graded (u), even though their *C_C_* values are slightly greater than 1. The notation for these samples follows the same convention as for the CR(w) mixtures; for example, ‘SR(p)/42.1%’ denotes a poorly graded mixture of S(u) and R(u) that contains 42.1% rubber by weight.

### 2.2. Standard Methods

There are no universally accepted procedures for determining *ρ_min_* and *ρ_max_* in non-cohesive, permeable soils. This is evident by the considerable variability among the selected national standards shown in Table A1, Table A2, Table A3, Table A4, Table A5, Table A6, Table A7, Table A8, Table A9, Table A10, Table A11 and Table A12 in Appendix A, which summarise the methods specified in ASTM D 4253 [58] and ASTM D 4254 [59], BS 1377 [60], DIN 1826 [61], NF P 94 059 [62], JGS 0161 [63], and PN-88/B-04481 [64], commonly used in the USA, United Kingdom, Germany, France, Japan, and Poland, respectively. It should be noted that PN-88/B-04481 has been withdrawn in Poland, but it has not been superseded by any subsequent document. The listed documents differ in many respects, including the allowable grain size range, required sample weight, type of apparatus, and the specific procedures for mould filling, load application, and soil compaction. These standards further vary in the required number of test repetitions and in the methods used to process results and are presented with diverse levels of procedural detail. Some standards include specific cautionary notes relevant to this study. For example, ASTM D 4254 [3] warns that, when determining *ρ_min_*, soil compaction, bulking, and particle segregation must be avoided. It also notes that water content can significantly influence *ρ_min_*. BS 1377 [60] states that compaction using a vibrating hammer is not applicable to soils susceptible to crushing; a restriction shared by DIN 18126 [61]. Similar to ASTM D 4254, the German standard excludes soils that are highly prone to segregation, indicating that non-uniform soils with rounded grains, *C_U_* > 12, and *d*_100_ > 31.5 mm present a high risk of segregation.

In most standards, *ρ_min_* is determined by placing oven-dry soil in a cylindrical container with a volume ranging from 113 to 14,200 cm^3^, depending on the particle size distribution of the material being examined. Three main methods are used to achieve the loosest possible arrangement of grains: (i) carefully placing the soil into the mould with a scoop or small shovel [59,60,61], (ii) pouring it through a funnel or similar device that is gradually raised above the mould base [59,61,63,64], or (iii) initially placing the soil in a measuring cylinder and then inverting it so that the grains are allowed to fall freely [59,60]. Depending on the standard, this procedure is repeated from 2 to 9 times, and the reported result is either the lowest measured value or the arithmetic mean of three mutually consistent measurements.

Determination of *ρ_max_* involves inducing mechanical vibrations to the soil inside a mould, typically the same mould used for *ρ_min_*. This can be done with a vibration fork [61,64], vibrating hammer [60], wooden hammer [63] or vibrating table [58,61] using a wide range of vibration amplitudes, frequencies (25–60 Hz), and durations (1–12 min). The specimens are tested either oven-dried or fully saturated. During vibration, the specimens remain unloaded [63] or are subjected to vertical stresses ranging from 1.3 kPa [61,64] up to 46 kPa [60]. This determination is generally carried out twice. Identical to *ρ_min_*, the reported *ρ_max_* is either the highest measured value or the arithmetic mean of the measurements.

The observed variability in these standard procedures can significantly affect the results obtained. This implies that *ρ_min_* and *ρ_max_* should not be regarded as fixed, unique material constants for a given soil; therefore, the specific details of the applied procedure should always be reported. ASTM D 4253 [58] and ASTM D 4254 [59] explicitly note that the absolute minimum and maximum densities are not necessarily achieved by the standard test methods and that the outcomes depend on operator expertise as well as on the adequacy of the equipment and facilities. In light of this, several different procedures were employed in this research to determine those yielding the loosest and densest attainable particle arrangement in a mould whose dimensions were as close as possible to those of the specimens intended for subsequent geotechnical testing (70 mm in diameter and 140 mm in height).

### 2.3. Applied Methods

In this research, the minimum bulk density was determined using three different procedures: (A) gently introducing the material into a mould with a funnel, spoon, or scoop; (B) filling the mould to about one-half to three-quarters of its height, then slowly inverting it several times, setting it upright again, and finally filling the remaining volume with a spoon; or (C) slowly inverting a 1 L plastic measuring cylinder, graduated in 10 mL increments and 62 mm in diameter, filled with soil, and then carefully placing it on a flat surface. For methods (A) and (B), surplus soil was removed with a straightedge, and any larger voids were filled with finer particles. Each *ρ_min_* measurement was performed at least twice. In method (C), before the cylinder was placed on the table, it was slowly rotated to obtain a levelled upper surface of the material and to allow for accurate reading of the volume scale. For each soil batch, the sequence of inverting the cylinder and reading the volume was repeated until three identical readings were obtained, and only that value was recorded. The final reported value was the lowest of all measurements.

Given the specimen dimensions needed to evaluate the mechanical and dynamic properties of the mixtures, it was decided to use, for methods (A) and (B), an existing metal cylindrical mould with an internal diameter and height equal to 71 and 126 mm, respectively, corresponding to a volume of 497 cm^3^. This mould was manufactured in accordance with PN-88/B-04481 [64] and complies with the requirements for special moulds specified in ASTM D 4253 [58] and ASTM D 4254 [59].

**Figure 5:**
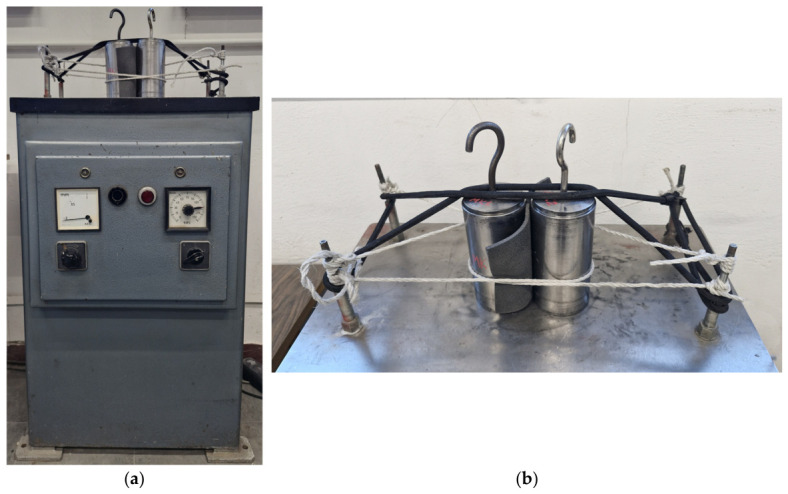
The vibrating table (**a**) and two moulds filled with soil during vibration (**b**); photo: M. Kowalska.

To obtain the maximum bulk density, the material was placed into the mould in 4–5 layers. Each layer was manually compacted with a tamper, taking sufficient care not to crush the RCA particles. Subsequently, a metal piston with the same diameter as the mould, weighing approximately 532 g and exerting a vertical stress of 1.3 kPa, was positioned on top of the specimen and firmly pressed down by hand in a static manner. The entire assembly was then fixed to a vibrating table using nylon and rubber strings (see Figure 5) and subjected to 50 Hz vibrations for 30–60 s. The single vibration amplitude *A* was increased stepwise (0.1/0.4/0.6 mm) after no additional piston settlement was observed in two successive readings. At the end of the procedure, the piston was rotated and again pressed statically by hand to prevent any interlocking. For specimens with a high rubber content, an additional period was provided to allow the material to fully recompress prior to recording the final specimen volume. Each measurement was taken twice. The reported *ρ_max_* value was the highest of all the calculated densities.

Badarayani (2021) [65] showed that adding a small amount of water into soil–rubber mixtures can reduce segregation. Therefore, the triaxial and resonant column tests previously mentioned were planned to be carried out on specimens with a water content of 5%. This *w* value was also used in earlier studies on sand–rubber mixtures [15,16,66]. In the present work, *ρ_min_* and *ρ_max_* were thus determined for both air-dry (*w* < 1%) and moist (*w* ≈ 5%) specimens. The measurements for S(u), R(u) and SR mixtures were partly taken from [67].

The results were further compared with void ratios obtained after impact compaction in a Proctor test and after controlled static loading. The Proctor tests were performed on selected specimens in accordance with standard PN-88/B-04481 [64] using a 1 L mould and normal compaction energy of 0.6 J/cm^3^. The relationship between water content and dry density was established, with particular emphasis on the points at *w* < 1% and *w* ≈ 5%; the corresponding dry densities and void ratios are denoted as *ρ_d,Pr_* and *e_Pr_*, respectively. The optimum water contents *w_opt_* and the maximum dry densities *ρ_d,max,Pr_* were also identified. The parameters *w_opt_* and *ρ_d,max,Pr_* for sand S(u), uniform rubber R(u) and SR mixtures were taken from previous investigations of these materials by Banzibaganye [50] (‘sand S2′), Kowalska [68] (‘specimen B’), and Ziemba [67]. The behaviour under static loading was examined for air-dry specimens using a simplified approach, namely in a shear box with an internal width of 100 mm and a height of 39 mm. The material was placed into the box in three layers and compacted with a tamper to achieve *I_D_* ≈ 70%. The initial states of the S(u), R(u) and SR specimens were consistent with [15,16], i.e., the sand and rubber were compacted to *I_D_* ≈ 80%, while the initial void ratios of sand–rubber mixtures varied linearly with the rubber content by volume *Χ_V_*. The dry densities and void ratios at vertical stresses *σ* = 25, 50, 100, and 200 kPa were determined; these are referred to as *ρ_d,σ_* and *e_σ_*, respectively. A summary of all tests performed in this study and sources from the literature for selected data is presented in Table 4.

## 3. Results and Discussion

### 3.1. Single-Material Samples

**Figure 6:**
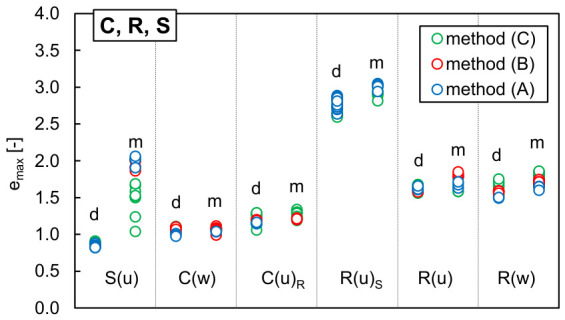
Influence of the method used to determine the maximum void ratio *e_max_* in the dry (‘_d’) and moist (‘_m’) single-material samples: sand (S), RCA (C), and TDA (R).

**Figure 7:**
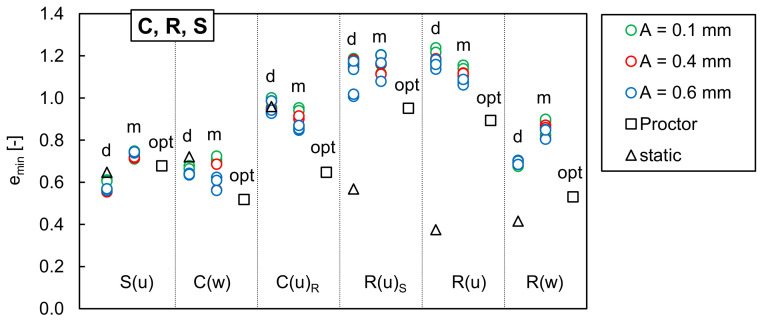
Influence of the single amplitude of vibration *A* used to determine the minimum void ratio *e_min_* in single-material samples: sand (S), RCA (C), and TDA (R), compared with the results of static loading (*σ* = 200 kPa) and Proctor compaction; the descriptions ‘d’, ‘m’ and ‘opt’ denote the specimen’s water content *w*: less than 1%, about 5% and optimum, respectively.

Figure 6 presents the maximum void ratios determined for the single-material samples using procedures (A), (B) and (C). In turn, Figure 7 shows the minimum void ratios recorded at progressively increasing vibration amplitudes for these samples. The lowest values of *e_min_* and the highest values of *e_max_* are reported in Table 5, along with the void ratios *e_Pr_* and *e_σ_* obtained respectively after impact compaction in the Proctor test and after controlled static loading. The corresponding dry densities are provided in Table A13 in Appendix B.

**Figure 8:**
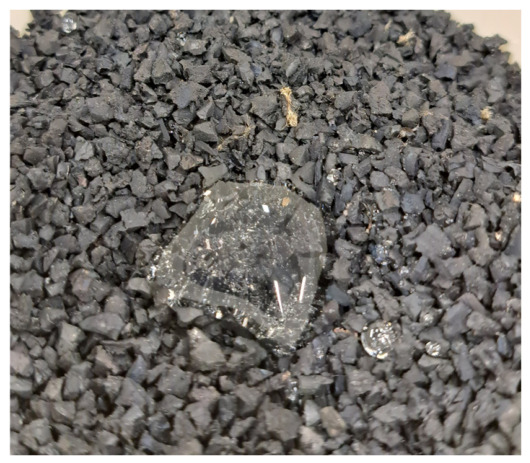
Hydrophobic effect of rubber: a water droplet resting on the surface of the R(u) specimen; photo: M. Kowalska.

From Figure 6, it can be noticed that the scatter in the *e_max_* values for moist fine sand, S(u), was much larger than for other single-material samples. Increasing the water content resulted in highly overestimated *e_max_* values; when the water content rose from 0% (dry) to ~5% (moist), the highest measured *e_max_* became 14 to 125% greater than the maximum value obtained for the air-dry specimen. This is likely due to capillary water holding fine particles against gravity and creating random air pockets between aggregates. This effect was more pronounced when the material was carefully placed into the mould using a spoon (method A) and less significant when the sand aggregates were allowed to fall freely into a plastic cylinder (method C). Although the particle size distribution of the moist R(u)_S_ was identical to that of S(u), the maximum void ratio obtained for that specimen was only 6% greater than in the dry R(u)_S_. This is presumably due to the hydrophobic nature of rubber, which is clear when a water droplet is placed on the specimen surface, as illustrated in Figure 8. In other moist single-material specimens, the maximum recorded void ratios were up to 10% higher than in the air-dry ones. These observations support the comments given in ASTM D 4254 [3] regarding the influence of water content on determining *ρ_min_* and explain why all standard procedures require *e_max_* to be measured on dry soil.

Except for R(u)_S_, in dry specimens, the highest *e_max_* values were obtained with the graduated cylinder method. The maximum void ratios of dry TDA were greater than those of RCA, and for both materials, smaller median particle sizes corresponded to smaller maximum *e_max_* values. Among all tested materials, the dry uniform sand exhibited the lowest *e_max_*.

As shown in Figure 7, except for S(u), all single-material samples reached their lowest *e_min_* at the end of the full vibration cycle, at a single amplitude of 0.6 mm. The effect of increased water content depended on the material type: in S(u), R(u)_S_ and R(w), additional water reduced the efficiency of vibration, while in C(w), C(u)_R_ and R(u), introducing some moisture made it possible to obtain denser specimens. To interpret this behaviour, it is useful to compare the vibration results with those from other densification methods—see Figure 7, Table 5 and Table A13 in Appendix B. The discrepancy between the *e_min_* values obtained from vibration, impact compaction, and static loading is substantial—smallest in sand and largest in rubber granulates—highlighting the limitations of the standard vibration procedure when applied to alternative materials.

**Figure 9:**
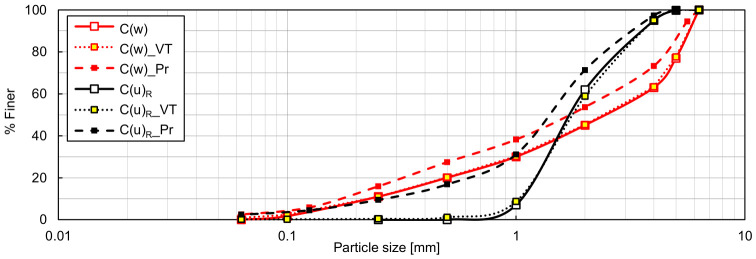
Particle size distributions of the C(w) and C(u)_R_ specimens: initial, after determination of *e_min_* using a vibration table (_VT) and after Proctor compaction test (_Pr).

In RCA, regardless of grading characteristics, the highest *ρ_d,max_* and the lowest *e_min_* were consistently achieved through Proctor compaction at the optimum water content, of approximately 17%. This is likely due to the rough surface texture of RCA particles, whose interparticle friction must be partially overcome by adding an appropriate amount of water. Additionally, the impact loading promotes crushing of some weaker particles or detachment of the residual mortar from the grain surfaces, as evidenced by the evolution in particle size distribution of the RCA samples after Proctor compaction. Figure 9 compares the initial grading curves of C(w) and C(u)_R_ specimens with those obtained after densification using the vibration table and after Proctor compaction. Vibration induced only negligible changes in grading, whereas Proctor compaction produced a noticeable shift in the curves towards the finer fractions. The impact loading caused crushing of coarser particles, reducing the median particle size *d*_50_ in the C(w) and C(u)_R_ specimens from 2.45 to 1.70 mm and from 1.68 to 1.40 mm, respectively. The coefficient of uniformity *C_U_* determined for C(w) after Proctor compaction remained above 15, but the coefficient of curvature *C_C_* decreased to 0.84, falling below the lower threshold for well-graded classification; the overall particle size range was preserved, but the grading quality slightly deteriorated. In C(u)_R_, the grading characteristics significantly improved after impact compaction (*C_U_* increased to 6.19 and *C_C_* to 2.30), resulting in a material classifiable as medium-graded with a more favourable particle size distribution. In both cases, the newly generated fine particles gradually filled the intergranular voids, causing a more effective densification of RCA when compared to vibration.

In the uniform fluvial sand S(u), where grains are rounded, smoother, and more resistant to particle breakage, vibration produced the densest packing. Oven-drying removed capillary forces between grains, allowing them to move independently. Due to the energy-dissipating characteristics of rubber, vibration was the least effective method for densifying TDA. In most cases, extended vibration even loosened the structure previously formed by manual pressing. Impact loading at the optimum water content produced slightly better densification, but a significantly lower void ratio was obtained under static loading of the specimen. Although the specimens partially rebounded after unloading, rubber particles tended to deform and become permanently interlocked far more effectively than the rigid concrete aggregate or sand grains. This was corroborated by the noticeable difficulty in extracting compressed TDA samples from the mould or shear box. Since the minimum void ratio in TDA decreases with increasing static vertical stress, it can be inferred that at sufficiently high loads, *e_min_* may approach zero.

The influence of grading characteristics on the boundary void ratios of RCA and TDA can be evaluated by comparing the following samples: C(w) with C(u)_R_, and R(u)_S_ with R(u) and R(w). As anticipated, after vibration and Proctor compaction, the well-graded RCA and TDA exhibited markedly lower void ratios than the uniformly graded materials, whereas the *e_min_* values of the two uniformly graded rubber granulates were quite similar. Under a static load of 200 kPa, the well-graded RCA also developed a denser particle structure than the uniformly graded specimen. For rubber granulates, the effect of *C_U_* and *C_C_* on *e_min_* under static loading appears to be less significant than the influence of particle size, though this observation requires further investigation.

### 3.2. Soil-Rubber Mixtures

**Figure 10:**
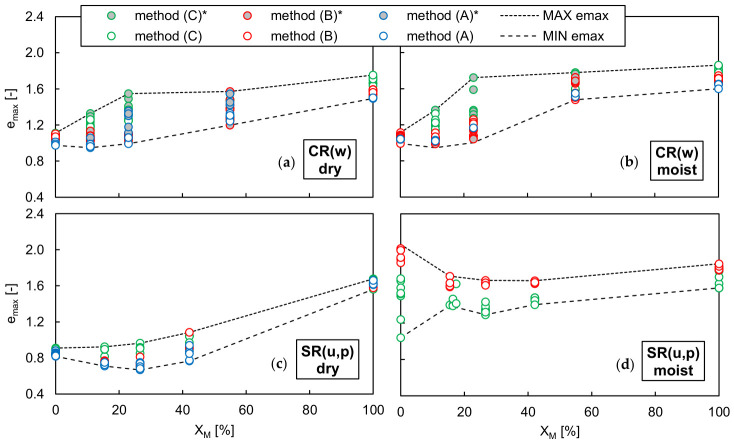
Influence of the method selected for determining the maximum void ratio *e_max_* in well-graded dry (**a**) and moist (**b**) RCA-TDA (CR(w)) mixtures and in uniformly/poorly graded dry (**c**) and moist (**d**) sand–TDA (SR(u,p)) mixtures as a function of the rubber content by weight *Χ_M_*; the asterisk marks the results for the samples with *η* ≠ 1.

**Figure 11:**
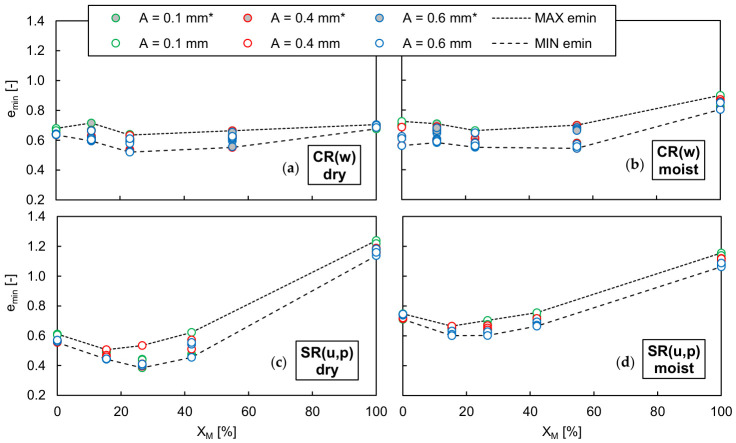
Influence of the vibration amplitude *A* on the minimum void ratio *e_min_* in well-graded dry (**a**) and moist (**b**) RCA-TDA (CR(w)) mixtures and in uniformly/poorly graded dry (**c**) and moist (**d**) sand–TDA (SR(u,p)) mixtures as a function of the rubber content by weight *Χ_M_*; the asterisk marks the results for the samples with *η* ≠ 1.

**Figure 12:**
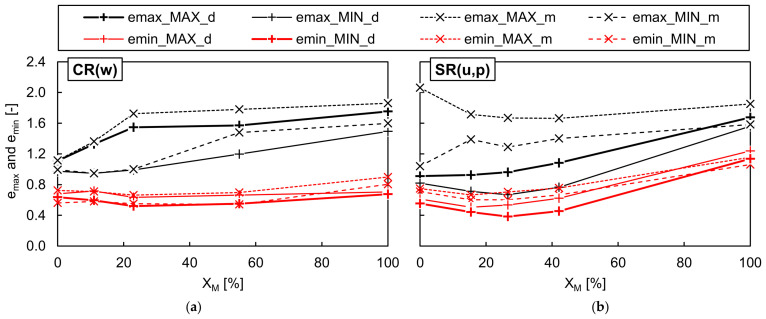
The highest (‘_MAX’) and lowest (‘_MIN’) values of the maximum and minimum void ratios obtained in dry (‘_d’) and moist (‘_m’) well-graded RCA-TDA (CR(w)) (**a**) and in uniformly/poorly graded sand–TDA (SR(u,p)) (**b**) mixtures as a function of the rubber content by weight *Χ_M_*.

Figure 10 and Figure 11 show the maximum and minimum void ratios of the dry and moist well-graded RCA-TDA mixtures, as well as the uniformly/poorly graded sand–TDA mixtures, obtained using various test methods and plotted as a function of the rubber content by weight. The dashed lines in the graphs represent envelopes defined by the highest and lowest values, which were then combined and are jointly illustrated in Figure 12. In Figure 10 and Figure 11, the asterisks mark the results for the RCA-TDA mixtures prepared by substituting selected particle size fractions of well-graded RCA with TDA (method M2). The dry densities for all mixtures tested are reported in Table A14 and Table A15 of Appendix B.

**Figure 13:**
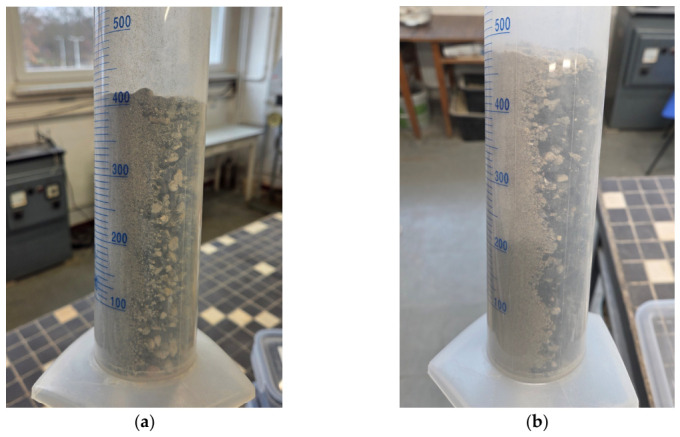
Segregation of the smaller and larger particles in the samples CR(w)/11% (**a**) and CR(w)/23%/>5.0 mm (**b**) after rotation in a graduated cylinder; photo: B. Bdzionek.

As illustrated in Figure 10a,b, for all dry and moist CR(w) samples, method C consistently yielded the highest *e_max_* values, while method A yielded the lowest. Since a transparent graduated cylinder was used for method C, it was possible to observe that the slow rotation of the cylinder caused segregation of the particles along the height of the sample—see Figure 13—which may be responsible for the higher void ratios. The effect was well visible in the RCA-TDA mixtures due to the difference in colour of the components, but it also occurred in the single materials. The segregation consisted of the separation of the grains according to their size (larger from smaller) and then according to their weight (rubber from concrete or sand). It may be assumed that this effect was less pronounced when method A was used. Overall, the scatter in the measurements was greater than that obtained for the single materials, C(w) and R(w). The greatest discrepancy between the highest and lowest *e_max_* values was observed in the moist sample C(w)/23%/>5.0 mm, where the difference amounted to 42% of the highest *e_max_*. In comparison with the mixtures produced by combining well-graded RCA and well-graded TDA (method M1), the mixtures where rubber was present only in the coarser fraction exhibited higher *e_max_* values (by 2–36%), while the mixture in which rubber was present in the finest fractions showed lower *e_max_* values (by 12–15%). When focusing solely on the CR(w) mixtures with *η* = 1, it can be reasonably assumed that, regardless of the testing procedure used, *e_max_* increases almost linearly with increasing rubber content, as illustrated by the black lines in Figure 12a.

The difference between the highest and lowest *e_min_* values obtained for the CR(w) samples during specimen vibration varied from 2.5% (for moist CR(w)/11%/≤0.25 mm) to 23% (for dry CR(w)/23%) of the lowest *e_min_*. No systematic effect of the mixture preparation procedure, vibration amplitude, or water content was observed, as shown in Figure 11a,b. It may be inferred that, for *w* ≤ 5% and *Χ_M_* ≤ 60%, the minimum void ratio of the CR(w) mixtures does not depend on the rubber content and corresponds to the value achieved in C(w), i.e., approximately 0.6; refer to the red lines in Figure 12a.

As shown in Figure 10c,d, for the sand–TDA mixtures, the use of the plastic graduated cylinder was the most effective method for achieving the highest *e_max_* values in dry specimens. However, similar to S(u) and R(u), it resulted in the lowest *e_max_* values when the specimens were moist. Focusing only on the more reliable results obtained for dry samples, it can be observed that there is a rubber content, *Χ_M_* ≈ 30%, at which the maximum void ratio becomes smaller than that of both sand alone and rubber alone.

The effect of rubber content on the minimum void ratio of the sand–TDA mixtures is also evident in Figure 11c,d. Under vibration, the mixtures achieved their lowest *e_min_* at *Χ_M_* ≈ 30%, without a clear dependence on vibration amplitude, yielding a parabolic relationship between *e_min_* and *Χ_M_*. This behaviour can be attributed to the migration of finer, denser sand particles into the voids between rubber particles in the lower parts of the mould. This phenomenon, occurring in binary mixtures of contrasting particle sizes, was described previously by Lee et al. (2007) [69] and Kim and Santamarina (2008) [70]. As observed for the sand-only case, *e_min_* values obtained from the moist specimens were higher (by up to 57% of the lowest *e_min_*) than those from the dry specimens. The observed flattening of the *Χ_M_*–*e* curve is caused by reduced particle segregation upon the introduction of water into the mixture. For moist sand–TDA mixtures, *e_min_* exhibits a notably weaker dependence on *Χ_M_* compared to dry conditions. This is consistent with the role of capillary forces, which develop at contact between sand particles and the rubber surface in the presence of water, partially restraining the relative sliding and migration of sand grains, a mechanism previously reported for sand–rubber mixtures subjected to vibration [65]. As a result, the migration of sand into the intergranular voids of the rubber skeleton is suppressed, leading to a more uniform void ratio across the range of rubber contents investigated.

**Figure 14:**
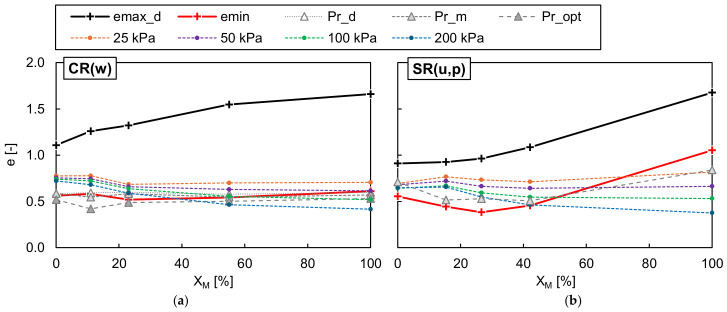
The maximum and minimum void ratios of well-graded RCA-TDA (CR(w)) (**a**) and uniformly/poorly graded sand–TDA (SR(u,p)) (**b**) mixtures as a function of the rubber content by weight *Χ_M_*, compared with the results of static loading at *σ* = 25/50/100/200 kPa on dry specimens and impact loading on mixtures at *w* < 1% (Pr_d), *w* ≈ 5% (Pr_m) and *w* = *w_opt_* (Pr_opt).

**Figure 15:**
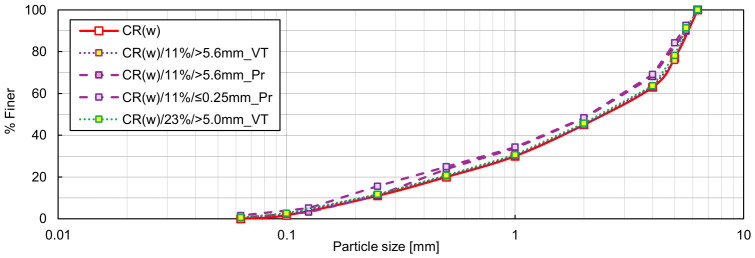
Particle size distributions of selected CR(w) mixtures: initial, after determination of *e_min_* using a vibration table (_VT) and after Proctor compaction test (_Pr).

Figure 14 compares the highest *e_max_* (considering only dry samples) and lowest *e_min_* for RCA-TDA mixtures (limited to those with *η* = 1) and sand–TDA mixtures with void ratios obtained under impact and static loading. It can be observed that for the CR(w) samples, Proctor compaction at *w_opt_* proved more effective for soil densification than vibration table compaction, resulting from the particle breakage of the RCA particles. The damping properties of rubber mitigate this effect in RCA-TDA mixtures compared to the single-material RCA samples, as can be inferred by comparing the particle size distributions of selected CR(w) specimens before and after vibration and impact compaction (Figure 15) with the corresponding results for RCA (Figure 9). On the other hand, for *Χ_M_* values greater than 40%, the lowest *e_min_* values were achieved under static loading with vertical stresses exceeding 100 kPa. For all SR(u,p) samples, neither Proctor compaction nor static loading yielded *e_min_* values lower than those achieved by vibration. Nevertheless, for *Χ_M_* > 50%, sand–rubber mixtures are expected to exhibit increasingly rubber-like behaviour. This is supported by the observation that, for R(u) samples, static loading at vertical stresses as low as 25 kPa produced significantly denser particle arrangements than either vibration table or impact compaction.

### 3.3. Comparison with Literature Data

**Figure 16:**
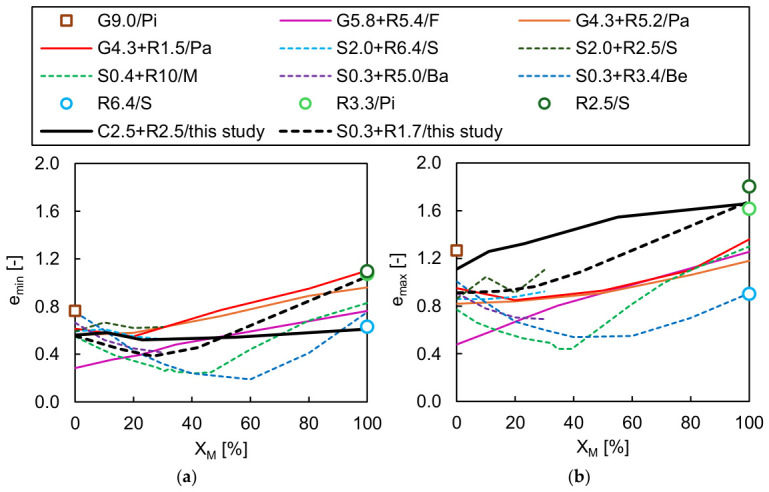
Effect of the rubber content by weight *Χ_M_* on the minimum (**a**) and maximum (**b**) void ratios of the RCA-TDA and sand–TDA mixtures evaluated in this study, in comparison with results reported in the literature.

In Figure 16, the void ratio boundaries for the RCA-TDA and sand–TDA mixtures from Figure 14 are compared with the results from other studies on similar materials. The symbols used in the figure encode the material type—C for RCA, R for TDA, S for sand, G for gravel—together with its *d*_50_ (in mm) and the identifier of the literature reference: Ba for Banzibaganye (2022) [50], Be for Benjelloun et al. (2022) [45], F for Fiamingo et al. (2025) [47] (or Fiamingo and Chiaro (2025) [48]), M for Mashiri (2014) [43], Pa for Pasha et al. (2019) [44], Pi for Pitilakis et al. (2024) [46], and S for Srivastava et al. (2026) [49]. It should be highlighted that the cited literature data relate to mixtures in which both the soil and rubber fractions are uniformly graded (*C_U_* = 1.0–2.7, *C_C_* = 0.9–2.2); a single exception is the research by Fiamingo et al. (2025) [48], who examined mixtures of well-graded sandy gravel (*C_U_* = 27 and *C_C_* = 2.2) combined with uniform TDA (*C_U_* = 1.9 and *C_C_* = 1.5). The reported values were derived using different testing procedures: (i) Banzibaganye (2022) [50] followed DIN 1826 [61] but employed two moulds of different sizes, with *D_m_* = 71 or 99 mm and *V_m_* = 444 or 858 cm^3^, to determine *ρ_min_* and *ρ_max_*, respectively, without specifying the vibration equipment; (ii) Benjelloun et al. (2022) [45] applied standard FN P 94 059 [62]; (iii) Fiamingo et al. (2025) [48] used ‘method A’ of ASTM D 4254 [59] and a special mould with *V_m_* = 942 cm^3^ to evaluate *ρ_min_*, whereas *ρ_max_* was obtained from Proctor compaction tests carried out according to ASTM D698 [71] (‘method C’); (iv) Mashiri (2014) [43] tested oven-dried material and adopted Australian standard AS 1289.5.51 (1998) [72], which is analogous to ASTM D 4254 [59] and ASTM D 4253 [58], using a special mould with a volume of *V_m_* = 1000 cm^3^; (v) Pasha et al. (2019) [44] followed JGS 0162 [73], utilising a mould with a volume of *V_m_* = 21,106 cm^3^, a bowl to deposit the material as loose as possible, and a vibrating hammer applying frequency of 50 Hz to determine *ρ_max_*; (vi) Pitilakis et al. (2024) [46] implemented ASTM D 4254 [59] and ASTM D 4253 [58] but shared no details of the applied procedures; (vii) Srivastava et al. (2026) [49] applied ASTM D 4254 [59] to determine *ρ_min_* of sand and sand–rubber mixtures and ASTM D 4253 [58] together with Proctor compaction [71] to obtain *ρ_max_* of sand and sand–rubber mixtures, respectively; however, neither the exact standard procedures employed nor the methods applied for testing rubber powder and rubber granulate were specified. It should be noted that the applied standards or procedure details in the studies described in [46,48] were obtained through private scholarly correspondence.

The considerable scatter among the literature results shown in Figure 16 can be attributed not only to differences in the particle size distributions of the soil and rubber components but also to inconsistencies in the testing methods employed.

The parabolic form of the *Χ_M_*–*e_min_* and *Χ_M_*–*e_max_* curves for the SR(u,p) samples is consistent with findings reported in the literature for mixtures consisting of materials with markedly contrasting gradations, where *η* ≫ 1 and *η* ≪ 1. In contrast, the CR(w) results exhibit the same trend as those reported in [44,48] for mixtures with 0.9 < *η* < 1.2, namely an approximately linear correlation between rubber content and the limiting void ratios. The nearly horizontal *e_min_* boundary observed for the well-graded RCA-TDA mixtures can be explained by the identical grading of the concrete and rubber aggregates. In such a material, the voids between coarser grains are filled equally by the finer grains of either rubber and/or concrete located in the immediate vicinity, resulting in a homogeneous behaviour with no tendency for segregation. It should be noted that the dilation of pure TDA specimens during vibration, observed in this study, was similarly documented by Mashiri (2014) [43] for tyre chips (6/8/20 mm) and consistent with the findings of Edil and Bosscher (1994) [8], who highlighted the limited effectiveness of vibration in pure tyre chips.

## 4. Conclusions

This study investigated the minimum and maximum void ratios of recycled concrete aggregate, sand, tyre-derived aggregate, and their mixtures across varying particle size distributions, grading characteristics, rubber contents, and water contents. The results provide new insights into the packing behaviour of deformable granular geomaterials and address a significant knowledge gap concerning RCA and well-graded RCA-TDA mixtures. The findings emphasise the need of carefully selecting and precisely documenting the research procedures used for the determination of *e_max_* and *e_min_* or *ρ_d,max_* and *ρ_d,min_* to minimise undesirable effects and obtain reliable and reproducible results.

The results confirm that particle characteristics (including density, shape, deformability, and grading) as well as sample water content have a significant effect on the boundary void ratios of granular materials. TDA exhibited significantly higher maximum void ratios than mineral soils due to their low particle density and high deformability, whereas well-graded RCA and sand achieved denser packing and correspondingly lower void ratios. The influence of water content was found to be material-dependent: capillary effects significantly increased the measured maximum void ratios in fine sand, whereas rubber-rich specimens exhibited negligible water content sensitivity, consistent with the hydrophobic nature of rubber particles. The cylinder inversion procedure yielded the largest void ratios for the majority of dry specimens; however, it also induced particle segregation. The effect of water content on *e_min_* was comparatively less pronounced in general, except for sand-only samples. As expected, after vibration and Proctor compaction, well-graded RCA and TDA reached significantly lower void ratios than their uniformly graded counterparts, while the *e_min_* values of the two uniformly graded rubber granulates were comparable.

For well-graded RCA-TDA mixtures with equal median particle sizes (*η* = 1), *e_max_* increased approximately linearly with rubber content, while *e_min_* remained nearly constant up to an *Χ_M_* of approximately 60%. This indicates that the mineral skeleton governs the densest packing state in well-graded mixtures until rubber becomes the dominant structural component. In contrast, uniform/poorly graded sand–TDA mixtures exhibited a parabolic dependence of both *e_min_* and *e_max_* on rubber content, with the minimum at *Χ_M_* around 30%, attributable to the migration of fine sand grains into the voids between coarser rubber particles.

The method of mixture preparation was shown to significantly influence the packing behaviour. The replacement of coarse RCA fractions with rubber increased *e_max_*, whereas the substitution of fine fractions decreased it, demonstrating that targeted fraction replacement can serve as a design tool for tailoring the density and compressibility of recycled geomaterials.

A key finding of this study is that conventional laboratory procedures cannot yield unique boundary void ratios for alternative materials, such as RCA, TDA and their mixtures. In RCA, this arises from the presence of residual mortar adhering to grain surfaces, which may detach during the densification process and progressively change the grading characteristics of the material. In TDA, the viscoelastic and deformable nature of rubber particles accounts for its superior densification achieved under static loading, indicating that the concept of a limiting/boundary minimum void ratio, developed for rigid granular soils, is not directly applicable to rubber-rich geomaterials. This has important implications for the use of relative density as a state parameter in rubber-containing soils.

This extensive study demonstrates that vibration-based densification is the most effective for sand and sand–rubber mixtures, Proctor compaction at optimum water content is the most effective for RCA and RCA-TDA with *Χ_M_* < 40%, and static loading is the most effective for TDA and RCA-TDA with *Χ_M_* > 40%; the latter results from the high deformation and interlocking of the rubber particles. Therefore, these are the recommended procedures for the determination of *e_min_* in the respective materials.

The results presented constitute the first systematic dataset of boundary void ratios for well-graded RCA-TDA mixtures, providing a basis for the preparation of reproducible specimens for static and dynamic geotechnical testing. The findings support the suitability of RCA-TDA mixtures as sustainable geomaterials in civil engineering applications, including seismic isolation systems and earthwork structures.

## Figures and Tables

**Table 1:** Composition, specific density, and grading characteristics of the single-material samples.

Sample	*Χ_M_* [%]	*ρ_s_* [g/cm^3^]	*d*_50_ [mm]	*C_U_* [–]	*C_C_* [–]
C(w)	0	2.71	2.45	15.7	1.21
C(u)_R_	0	2.71	1.68	1.87	0.87
S(u)	0	2.65	0.32	2.19	1.21
R(u)	100	1.20	1.68	1.87	0.87
R(u)_S_	100	1.20	0.32	2.19	1.21
R(w)	100	1.20	2.45	15.7	1.21

**Table 2:** Composition, specific density, and grading characteristics of the RCA-TDA mixtures.

Sample	C [mm]	R [mm]	*Χ_M_* [%]	*ρ_s,mix_* [g/cm^3^]	*d*_50_ [mm]	*C_U_* [–]	*C_C_* [–]	*d*_50*,r*_ [mm]	*d*_50*,s*_ [mm]	*η* [–]	*C_U,r_* [–]	*C_C,r_* [–]	*C_U,s_* [–]	*C_C,s_* [–]
CR(w)/11%/>5.6 mm	≤5.6	>5.6	11	2.36	2.45	15.7	1.21	5.94	2.00	3.0	1.06	1.01	13.9	1.15
CR(w)/11%	*	*	11	2.36	2.45	15.7	1.21	2.45	2.45	1.0	15.7	1.21	15.7	1.21
CR(w)/11%/≤0.25 mm	>0.25	≤0.25	11	2.36	2.45	15.7	1.21	0.15	3.10	0.05	1.76	0.94	8.30	1.04
CR(w)/23%/>5.0 mm	≤5.0	>5.0	23	2.07	2.45	15.7	1.21	5.60	1.52	3.7	1.13	0.97	11.4	1.02
CR(w)/23%	*	*	23	2.07	2.45	15.7	1.21	2.45	2.45	1.0	15.7	1.21	15.7	1.21
CR(w)/55%/>2.0 mm	≤2.0	>2.0	55	1.56	2.45	15.7	1.21	4.75	0.61	7.8	2.02	1.14	6.15	0.86
CR(w)/55%	*	*	55	1.56	2.45	15.7	1.21	2.45	2.45	1.0	15.7	1.21	15.7	1.21

* All subfractions composing the C(w) and R(w) samples, i.e., 0.063–6.3 mm.

**Table 3:** Composition, grading characteristics, and specific density of the sand–TDA mixtures.

Sample	S [mm]	R [mm]	*Χ_M_* [%]	*ρ_s,mix_* [g/cm^3^]	*d*_50_ [mm]	*C_U_* [–]	*C_C_* [–]	*d*_50*,r*_ [mm]	*d*_50*,s*_ [mm]	*η* [–]	*C_U,r_* [–]	*C_C,r_* [–]	*C_U,s_* [–]	*C_C,s_* [–]
SR(u)/15.4%	*	*	15.4	2.21	0.34	2.34	1.17	1.68	0.32	5.3	1.87	0.87	2.19	1.21
SR(u)/26.6%	*	*	26.6	1.97	0.36	2.50	1.11	1.68	0.32	5.3	1.87	0.87	2.19	1.21
SR(p)/42.1%	*	*	42.1	1.72	0.44	4.32	0.65	1.68	0.32	5.3	1.87	0.87	2.19	1.21

* All subfractions composing the S(u) and R(u) samples.

**Table 4:** Summary of performed tests and other data sources *.

No.	Sample	at *w* < 1%				at *w* ≈ 5%			at *w_opt_*
		*ρ_d,min_*	*ρ_d,max_*	*ρ_d,Pr_*	*ρ_d,σ_*	*ρ_d,min_*	*ρ_d,max_*	*ρ_d,Pr_*	*ρ_d,max,Pr_*
1	C(u)_R_	●	●	●	●	●	●	●	●
2	C(w)	●	●	●	●	●	●	●	●
3	CR(w)/11%	●	●	●	●	●	●	●	●
4	CR(w)/11%/>5.6mm	●	●	●	●	●	●	●	●
5	CR(w)/11%/≤0.25mm	●	●	●	●	●	●	●	●
6	CR(w)/23%	●	●	●	●	●	●	●	●
7	CR(w)/23%/>5.0mm	●	●	●	●	●	●	●	●
8	CR(w)/55%	●	●	●	●	●	●	●	●
9	CR(w)/55%/>2.0mm	●	●	●	●	●	●	●	●
10	R(u)	●	[66]	○	●	●	[66]	[66]	[67]
11	R(u)_S_	●	●	●	◐	●	●	●	●
12	R(w)	●	●	●	●	●	●	●	●
13	S(u)	●	[66]	○	●	●	[66]	[66]	[50]
14	SR(p)/42.1%	●	[66]	○	●	●	[66]	[66]	[66]
15	SR(u)/15.4%	●	[66]	○	●	●	[66]	[66]	[66]
16	SR(u)/26.6%	●	[66]	○	●	●	[66]	[66]	[66]

*●—fully implemented, ◐—partially implemented, ○—not implemented, ‘[]’—from other source.

**Table 5:** Void ratios [dimensionless] of the single-material samples *.

Sample	at *w* < 1%							at *w* ≈ 5%			at *w_opt_*
	*e_min_*	*e_max_*	*e_Pr_*	*e*_25kPa_	*e*_50kPa_	*e*_100kPa_	*e*_200kPa_	*e_min_*	*e_max_*	*e_Pr_*	*e_Pr_*
C(w)	0.636	1.109	0.585	0.777	0.755	0.742	0.721	0.561	**1.114**	0.585	**0.519**
C(u)_R_	0.928	1.294	0.839	1.018	0.992	0.988	0.959	0.847	**1.338**	0.844	**0.648**
S(u)	**0.555**	0.911	-	0.694	0.681	0.641	0.648	0.711	**2.063**	0.710	0.678
R(u)	1.137	1.677	-	0.818	0.664	0.532	**0.376**	1.063	**1.851**	0.840	0.894
R(u)_S_	0.940	2.753	1.220	-	0.820	0.679	**0.569**	1.009	**2.911**	1.191	0.951
R(w)	0.611	1.661	0.596	0.706	0.616	0.519	**0.416**	0.710	**1.763**	0.570	0.531

* The bold red and green fonts indicate the sample’s highest and lowest void ratios, respectively.

## Data Availability

The original data presented in the study are openly available in RepOD at https://doi.org/10.18150/GEKCMG.

## Abbreviations

The following abbreviations are used in this manuscript:

(g)	Gap graded
(p)	Poorly graded
(u)	Uniformly graded
(w)	Well-graded
*A*	Single amplitude of vibration, mm
C	Recycled concrete aggregate
*C_C_*	Coefficient of curvature, dimensionless
*C_U_*	Coefficient of uniformity, dimensionless
*d*_50,*r*_	Median size of rubber (TDA) particles, mm
*d*_50,*s*_	Median size of soil (S, Gr or RCA) particles, mm
*D_m_*	Internal diameter of mould, m
*D_r_*	Relative density/density index, %
*D_s_*	Internal diameter of the spout of the pouring device or funnel, m
*D_t_*	Internal diameter of tube, m
*dx*	Maximum size of the x% of the sample by weight, %
*e_max_*	Maximum void ratio, dimensionless
*e_min_*	Minimum void ratio, dimensionless
*e_σ_*	Void ratio at the vertical stress *σ*, dimensionless
*f*	Frequency of vibration, Hz
G	Gravel
*I_D_*	Relative density/density index, %
*L_s_*	Length of the spout of the pouring device or funnel, m
M1, M2	Methods used to prepare rubber–soil mixtures
*m_r_*	Mass of rubber skeleton, g
*m_s_*	Mass of soil skeleton, g
R	Tyre-derived aggregate
RCA	Recycled concrete aggregate
S	Sand
TDA	Tyre-derived aggregate
*V_m_*	Volume of mould, m^3^
*V_p_*	Volume of pouring device or funnel, m^3^
*V_t_*	Volume of tube, m^3^
*w*	Water content, %
*w_opt_*	Optimum water content, %
*η*	Ratio of the median sizes of rubber and soil particles, =*d*_50,*r*_/*d*_50,*s*_, dimensionless
*ρ_d,max_*	Maximum dry density, Mg/m^3^
*ρ_d,max,Pr_*	Maximum dry density from Proctor test, Mg/m^3^
*ρ_d,min_*	Minimum dry density, Mg/m^3^
*ρ_d,σ_*	Dry density at vertical stress *σ*, Mg/m^3^
*ρ_max_*	Maximum bulk density, Mg/m^3^
*ρ_min_*	Minimum bulk density, Mg/m^3^
*ρ_s_*	Specific density, Mg/m^3^
*ρ_s.a_*	Specific density of mineral soil, Mg/m^3^
*ρ_s.mix_*	Specific density of a mixture, Mg/m^3^
*ρ_s.r_*	Specific density of rubber, Mg/m^3^
*σ*	Vertical stress, kPa
*Χ**_M_*	Rubber content by weight, %
*Χ**_V_*	Rubber content by volume, %

## Appendix A

Table A1, Table A2, Table A3, Table A4, Table A5, Table A6, Table A7, Table A8, Table A9, Table A10, Table A11 and Table A12 present the methods of determining *ρ_min_* and *ρ_max_* according to standards ASTM D 4253 [58], ASTM D 4254 [59], BS 1377 [60], DIN 1826 [61], and PN-88/B-04481 [64]. The parameter *d_x_* denotes the maximum size of x% of the sample by weight, which often influences the choice of equipment and the testing procedure. Equipment details are described with parameters *V_y_* and *D_y_*, representing the volume and internal diameter of y, respectively, where *y* = *m* for mould, *t* for tube, and *p* for pouring device or funnel, while *D_s_* and *L_s_* denote the inner diameter and length of the spout of the pouring device or funnel.

**Table A1:** Methods of determining *ρ_min_* according to ASTM D 4254 [59].

Soil Grading	Main Equipment	Procedures to Obtain the Loosest Particle Arrangement (Mass and Volume)	
*d*_100_ ≤ 75 mm or 38.1 mm, *d*_70_ ≤ 31.5 mm, *d*_15_ ≥ 0.075 mm	Mould: *V_m_* = 14,200 cm^3^, *D_m_* = 279.4 mm; shovel or scoop	method A (preferred)	Oven-dry soil (34 kg) is placed into the mould using a shovel or scoop held just above the soil surface. Excess soil (up to 25 mm) is trimmed to level the top. Larger particles are removed manually. Minor projections of larger particles above the rim should roughly balance the larger voids below it.
*d*_100_ ≤ 19 mm, *d*_15_ ≥ 0.075 mm	Mould: *V_m_* = 2830 cm^3^, *D_m_* = 152.4 mm; scoop		Oven-dry soil (11 kg) is placed into the mould using a scoop held just above the soil surface. Excess soil (up to 25 mm) is levelled by trimming. Larger particles are removed using fingers.
*d*_100_ ≤ 9.5 or 4.8 mm, *d*_15_ ≥ 0.075 mm	Mould: *V_m_* = 2830 cm^3^, *D_m_* = 152.4 mm; pour. dev.: *V_p_* = (1.25–2) ∙ *V_m_*, *D_s_* = 25 or 13 mm, *L_s_* = 150 mm		Oven-dry soil (11 kg) is poured into the mould through the pouring device in a spiralling motion, maintaining approx. 13 mm distance from the soil surface. The excess soil (13–25 mm) is trimmed.
*d*_100_ ≤ 19 mm, 9.5 mm, or 4.8 mm, *d*_95_ ≤ 9.5 mm, *d*_15_ ≥ 0.075 mm	Special mould *: *V_m_* < 2830 cm^3^, *D_m_* = 70–100 mm; scoop, pour. dev.: *D_s_* = 25 or 13 mm, *L_s_* = 150 mm		Oven-dry soil (mass depends on *V_m_*) is placed in the mould using a scoop held just above the soil surface or poured in a spiralling motion, maintaining approx. 13 mm distance from the soil surface. The excess soil (13–25 mm) is trimmed. Larger particles are removed using fingers.
*d*_100_ ≤ 19 mm, *d*_15_ ≥ 0.075 mm	Mould and spoon, scoop, or pour. dev. like in method A; rigid thin-walled tube: *V_t_* = (1.25–1.3) ∙ *V_m_*, *D_t_* ≈ 0.7 ∙ *D_m_*	method B	The tube is inserted into the mould. Oven-dry soil (mass like for method A) is carefully placed with a spoon, scoop, or pouring device into the tube, up to 3–6 mm below its top. Next, the tube is quickly raised, allowing the soil to fill the mould. The excess soil (13–25 mm) is trimmed like in method A.
fine and medium sands; *d*_100_ ≤ 9.5 mm, *d*_90_ ≤ 2 mm, *d*_15_ ≥ 0.075 mm	Glass graduated cylinder with a stopper in the top: *V_m_* = 2000 ± 20 mL, *D_m_* = 75 mm	method C	1 kg of oven-dry soil is placed in the graduated cylinder. After closing it with a stopper, the cylinder is tipped upside down and then quickly returned to its original vertical position. The volume of the soil is recorded from the scale.
RESULT: The arithmetic mean of 3 consistent values of density from trials that agree within 1%.			

* A special mould is used in special studies when there is not enough soil to use the standard mould.

**Table A2:** Methods of determining *ρ_max_* according to ASTM D 4253 [58].

Soil Grading	Main Equipment	Procedures to Obtain the Densest Particle Arrangement (Mass and Volume)
*d*_100_ ≤ 75 mm, *d*_15_ ≥ 0.075 mm	Mould and pouring device like in ASTM D 4254 [59]; surcharge base plate and weights applying 13.8 kPa in total; guide sleeve; methods 1A and 1B: electromagnetic, vertically vibrating table; methods 2A and 2B: eccentric or cam-driven vertically vibrating table	Methods 1A and 2A: Oven-dry soil is placed in the mould in accordance with the appropriate procedure specified in ASTM D 4254 [59] for the given soil type. The sides of the mould may be tapped to help the soil settle. The surcharge plate is then positioned on top of the specimen. The mould with the guide sleeve is attached to the vibrating table, and the surcharge weight is lowered onto the plate. A double amplitude of 0.33 mm at 60 Hz or 0.48 mm at 50 Hz is applied for 8 or 12 min, respectively *. After vibration, the mass and height of the specimen are determined.
		Methods 1B and 2B: The soil is mixed with an amount of water sufficient to ensure specimen saturation during densification while preventing free water from accumulating in the mixing pan after approximately 30 min of soaking. The mould is attached to the vibrating table, which is then switched on. Within 5–6 min, the soil is placed into the mould in layers using a scoop or shovel. A small amount of free water should be visible on each layer; if not, additional water must be added. The vibration parameters (double amplitude and frequency) are adjusted to avoid excessive soil boiling or fluffing. At the end of this process, any excess water on the specimen’s surface is removed. The surcharge plate and weight are then placed on top of the specimen, and the entire assembly is vibrated following the same procedure as in methods 1A and 2A. After vibration, any remaining free water is removed. The mass, height, and water content of the specimen are then determined.
RESULT: The average of 3 consistent values from trials that agree within 2%.		

* Use of other, optimum, double amplitude of vibration is allowed as long as it is within the range 0.2–0.64 mm at 60 Hz or 0.3–0.94 mm at 50 Hz.

**Table A3:** Methods of determining *ρ_min_* according to BS 1377 [60].

Soil Grading	Main Equipment	Procedures to Obtain the Loosest Particle Arrangement (Mass and Volume)
sands; *d*_100_ ≤ 5 mm; *d*_10_ ≥ 0.063 mm	Glass graduated cylinder with a rubber bung or membrane: *V_m_* = 1000 ± 20 mL	Oven-dry soil (1 kg) is placed in the cylinder and closed with the bung or membrane. It is shaken to loosen the particles and inverted a few times. The cylinder is turned upside down until the material rests, then quickly turned right way up and carefully placed on a flat surface. The mean volume of the material is recorded from the scale to the nearest 10 mL. The procedure of shaking, inverting and volume reading is repeated at least 9 times.
gravels and sandy gravels; *d*_100_ ≤ 37.5 mm; *d*_10_ ≥ 0.063 mm	Mould (with extension ring): *V_m_* = 2305 cm^3^, *D_m_* = 152 mm; bucket; scoop	The oven-dry soil (of volume ≥ 3457.5 cm^3^) is mixed in a bucket. It is then poured steadily into the mould from the height of about 0.5 m for about 1 s. The extension ring is removed, and the excess soil is trimmed. Large particles are picked off manually; a cavity left by removal of a large particle should be filled where possible with a single smaller particle. The mass of the contents of the mould is measured. The soil is remixed and the procedure is repeated at least 9 times.
RESULT: The minimum of the density values from 10 readings.		

**Table A4:** Methods of determining *ρ_max_* according to BS 1377 [60].

Soil Grading	Main Equipment	Procedures to Obtain the Densest Particle Arrangement (Mass and Volume)
sands (more than 50% of fraction 0.06–2 mm); *d*_10_ ≥ 0.063 mm; not more than 10% of fraction 2–6.3 mm	Mould (with extension ring): *V_m_* = 1000 cm^3^, *D_m_* = 105 mm; external watertight container; scoop; tamper (≤2.5 kg); electric vibrating hammer (operating frequency 25–45 Hz)	Two oven-dry soil samples (3 kg each) are poured into two buckets with warm water, stirred and left for a couple of hours. The mould is placed into an external container. Both are filled with water up to a depth of 50 mm. The soil portions are added to the mould with a scoop; the quantity of the material should be such that it reaches about 1/3 of the mould’s height after compaction. Water is added to the external container until it reaches the same level as in the mould. The tamper is placed on the levelled soil surface. The material is compacted with the hammer for at least 2 min or until there is no significant decrease in the specimen’s height; a steady load of 35–46 kPa should be applied during compaction. The procedure is repeated for the second and third layers, ensuring that the soil surface remains submerged. The tamper is removed. The mould with soil is taken out of the container, free water is drained, and the extension ring is removed. The excess soil (max. 6 mm) is trimmed off. Any cavities left by the removal of coarse particles are filled with finer material. The soil surface is well pressed in. The compacted soil is extracted from the mould, oven-dried, and weighed. The whole procedure is repeated for the second batch of soil. If the obtained dry masses differ by more than 50 g, the test must be carried out using fresh soil samples.
gravelly soils; *d*_100_ ≤ 37.5 mm; not more than 30% of fraction 6.3–20 mm; *d*_10_ ≥ 0.063 mm	As above, except for: *V_m_* = 2305 cm^3^, *D_m_* = 152 mm, Tamper (≤3 kg).	As above, except for: The mass of the soil sample in one batch is 8 kg, Tthe time of vibration applied to each soil layer is equal to at least 3 min, The allowed difference between the dry masses of the compacted soil in two batches is 150 g.
RESULT: The maximum of the values obtained from two separate batches.		

**Table A5:** Methods of determining *ρ_min_* according to DIN 1826 [61].

Soil Grading	Main Equipment	Procedures to Obtain the Loosest Particle Arrangement (Mass and Volume)
*d*_100_ ≤ 5 mm (*C_U_* ≥ 3) or *d*_100_ ≤ 2 mm (*C_U_* < 3); *d*_5_ ≥ 0.06 mm; not more than 50% of fraction 0.06–0.2 mm	Mould: *V_m_* = 431 cm^3^, *D_m_* = 71 mm; funnel: *V_p_* ≥ 500 cm^3^, *L_s_* = 175 mm, *D_s_* = 12 mm when *d*_100_ ≤ 2 mm, *D_s_* = 25 mm when *d*_100_ ≤ 5 mm; winch	The oven-dried soil for which the densest particle arrangement was determined (see Table A6) is prepared. The funnel is centred on the mould bottom, filled with the soil, then lifted using the winch while maintaining contact with the soil surface. Excess soil is trimmed. The test is repeated twice.
*d*_100_ ≤ 10 mm (*C_U_* ≥ 3) or *d*_100_ ≤ 5 mm (*C_U_* < 3); *d*_15_ ≥ 0.06 mm	Mould: *V_m_* = 943 cm^3^, *D_m_* = 100 mm; funnel: *V_p_* ≥ *V_m_*, *L_s_* = 175 mm, *D_s_* = 12 mm when *d*_100_ ≤ 2 mm, *D_s_* = 25 mm when *d*_100_ ≤ 5 mm; winch	
*d*_100_ ≤ 63 mm (*C_U_* ≥ 6); *d*_15_ ≥ 0.06 mm	Mould: *V_m_* = 2209 cm^3^, *D_m_* = 150 mm; trowel	The oven-dried soil for which the densest particle arrangement was determined (see Table A6) is prepared. The material is poured from the trowel or shovel at a shallow angle, close to the surface; large grains are placed manually. Excess is trimmed to balance material above and below the rim. The test is repeated twice.
*d*_100_ ≤ 31.5 mm; *d*_15_ ≥ 0.06 mm	Mould: *V_m_* = 9817 cm^3^, *D_m_* = 250 mm; shovel	
RESULT: The arithmetic mean of the results from 3 trials.		

**Table A6:** Methods of determining *ρ_max_* according to DIN 1826 [61].

Soil Grading	Main Equipment	Procedures to Obtain the Densest Particle Arrangement (Mass and Volume)
*d*_100_ ≤ 5 mm (*C_U_* ≥ 3) or *d*_100_ ≤ 2 mm (*C_U_* < 3); *d*_5_ ≥ 0.06 mm; not more than 50% of fraction 0.06–0.2 mm	Mould with a filter plate, filter paper and a draining tube at the base: *V_m_* = 431 cm^3^, *D_m_* = 71 mm; vibration fork (960 g); top plate with a handle (500 g)	1 kg of oven-dry soil is prepared. About 1/5 of the specimen’s mass is filled into the mould, and enough water is added to completely submerge the soil layer. The material is compacted by 30 double blows of the vibration fork over 8–10 s. The striking fork should touch the cylinder at its lower end, approximately 30–60 mm from the fork tips. The procedure is repeated until the whole material is used. The water is pumped out through the draining tube, and the top plate is placed on the soil surface. The mould is hit with 5–6 double blows of the fork; the top plate is lightly tapped as well. The specimen’s height is measured.
*d*_100_ ≤ 10 mm (*C_U_* ≥ 3) or *d*_100_ ≤ 5 mm (*C_U_* < 3); *d*_15_ ≥ 0.06 mm	Mould: *V_m_* = 943 cm^3^, *D_m_* = 100 mm; extension ring; piston with spring and weight applying 10 kPa; vibrating table	At least 6 kg (or 2 kg if fraction < 0.6 mm dominates) of oven-dry soil is prepared. The mould is attached to the vibrating table. The soil is filled evenly and loosely into the mould and levelled. The extension ring is inserted on the top of the mould and clamped. The piston is lowered onto the soil surface. The specimen is vibrated for 5 min at a frequency of 50 Hz. Its height is measured. The test must be repeated at least twice with fresh soil. Soil already used for the compaction test must not be used again if it contains grains greater than 0.6 mm.
*d*_100_ ≤ 31.5 mm; *d*_15_ ≥ 0.06 mm	As above, except for: *V_m_* = 2209 cm^3^, *D_m_* = 250 mm	As above, except for: The mass of the soil to be prepared is 18 kg.
*d*_100_ ≤ 63 mm (*C_U_* ≥ 6); *d*_15_ ≥ 0.06 mm	As above, except for *V_m_* = 9817 cm^3^, *D_m_* = 150 mm	As above, except for: The mass of the soil to be prepared is 90 kg.
RESULT: The arithmetic mean of the results from 3 trials.		

**Table A7:** Methods of determining *ρ_min_* according to FN P 94-059 [62].

Soil Grading	Main Equipment	Procedures to Obtain the Loosest Particle Arrangement (Mass and Volume)
*d*_100_ ≤ 5 mm; *d*_12_ > 0.08 mm	Mould A: *V_m_* = 750 cm^3^, *D_m_* = 100 mm; funnel: *D_s_* = 12.5 mm; winch	Oven-dry soil (at least 7.5 or 25 kg for moulds A and B, respectively) is prepared. The funnel is centred on the mould bottom, filled with the soil, then lifted vertically using the winch, maintaining a distance from the soil surface not exceeding 5 mm (0 mm for sands). The excess soil is trimmed.
5 mm < *d*_100_ ≤ 10 mm; *d*_12_ > 0.08 mm	Mould B: *V_m_* = 2500 cm^3^, *D_m_* = 150 mm; funnel: *D_s_* = 25 mm; winch	
10 mm < *d*_100_ ≤ 31.5 mm; *d*_12_ > 0.08 mm	Mould B: *V_m_* = 2500 cm^3^, *D_m_* = 150 mm; funnel: *V_p_* ≥ 1500 cm^3^, *L_s_* = 175 mm; trowel	Oven-dry soil (at least 25 or 100 kg for moulds B and C, respectively) is prepared. The material is placed into the mould with a trowel or shovel, ensuring that its edge is close to the surface without touching it. The excess soil is trimmed.
31.5 mm < *d*_100_ ≤ 50 mm; *d*_70_ ≤ 31.5 mm; *d*_12_ > 0.08 mm	Mould C: *V_m_* = 10,000 cm^3^, *D_m_* = 250 mm; funnel: *V_p_* ≥ 1500 cm^3^, *L_s_* = 175 mm; shovel	
RESULT: The arithmetic mean of the results from at least 5 trials.		

**Table A8:** Methods of determining *ρ_max_* according to FN P 94-059 [62].

Soil Grading	Main Equipment	Procedures to Obtain the Densest Particle Arrangement (Mass and Volume)
*d*_100_ ≤ 5 mm; *d*_12_ > 0.08 mm	Mould A: *V_m_* = 750 cm^3^, *D_m_* = 100 mm; funnel: *D_s_* = 12.5 mm; extension ring; piston with spring and weight applying 10 kPa; vibrating table; gauge shim	After determining *ρ_min_*, the mould with loose soil is fixed to the vibrating table, an extension ring is attached, and the piston is placed on the soil surface. The specimen is vibrated for 8 min ± 15 s at a frequency of 50 Hz and double amplitude of 0.5 mm ± 0.1 mm. After vibrating, the piston and extension ring are removed, and the gauge shim is inserted. The sample’s height is the arithmetic mean of the measurements at 3 points on the gauge’s perimeter. The mass of the soil is determined after removing the gauge shim.
5 mm < *d*_100_ ≤ 10 mm; *d*_12_ > 0.08 mm	Mould B: *V_m_* = 2500 cm^3^, *D_m_* = 150 mm; funnel: *D_s_* = 25 mm; extension ring; piston with spring and weight applying 10 kPa; vibrating table; gauge shim	
10 mm < *d*_100_ ≤ 31.5 mm; *d*_12_ > 0.08 mm	Mould B: *V_m_* = 2500 cm^3^, *D_m_* = 150 mm; trowel; extension ring; piston with spring and weight applying 10 kPa; vibrating table; gauge shim	
31.5 mm < *d*_100_ ≤ 50 mm; *d*_70_ ≤ 31.5 mm; *d*_12_ > 0.08 mm	Mould C: *V_m_* = 10,000 cm^3^, *D_m_* = 250 mm; shovel; extension ring; piston with spring and weight applying 10 kPa; vibrating table; gauge shim	
RESULT: The arithmetic mean of the results from at least 2 trials.		

**Table A9:** Methods of determining *ρ_min_* according to JGS 0161 [63].

Soil Grading	Main Equipment	Procedures to Obtain the Loosest Particle Arrangement (Mass and Volume)
Sands: *d*_100_ < 2 mm; *d*_5_ > 0.075 mm	Mould: *V_m_* = 113.1 cm^3^, *D_m_* = 60 mm; paper funnel: *D_s_* = 12 mm	A minimum of 500 g of oven-dry soil is prepared. The funnel is positioned centrally on the bottom of the mould, filled with oven-dried soil, and then raised vertically at a uniform speed so that the mould is overfilled within 20–30 s while keeping a continuous flow and contact with the sample surface. The excess soil is trimmed.
RESULT: No minimum number of tests given.		

**Table A10:** Methods of determining *ρ_max_* according to JGS 0161 [63].

Soil Grading	Main Equipment	Procedures to Obtain the Densest Particle Arrangement (Mass and Volume)
Sands: *d*_100_ < 2 mm; *d*_5_ > 0.075 mm	Mould: *V_m_* = 113.1 cm^3^, *D_m_* = 60 mm; collar: 23 mm in height; wooden hammer: 30 mm in diameter	The collar is mounted on the mould. The sample used to determine *ρ_min_* is divided into ten portions and placed into the mould in successive layers. After each layer is added, the side of the mould is struck 100 times with the wooden hammer. A given point on the mould is hit 5 times within approximately 1 s, with a hammer swing of about 5 cm; then, the mould is rotated by 45–90° and the procedure is repeated. The soil in the 10th layer should overfill the mould. Once compaction is completed, the collar is removed and any excess material is trimmed off.
RESULT: No minimum number of tests given.		

**Table A11:** Methods of determining *ρ_min_* according to PN-88/B-04481 [64].

Soil Grading	Main Equipment	Procedures to Obtain the Loosest Particle Arrangement (Mass and Volume)
*d*_100_ ≤ 5 mm, *d*_90_ ≤ 2 mm	Mould *V_m_* = 500 cm^3^, *D_m_* = 70 mm; funnel	Oven-dry soil (600 cm^3^) is poured into the mould through a funnel, which is gradually raised as the container fills, maintaining the smallest possible distance from the soil surface. The excess soil is trimmed.
RESULT: The minimum from 5 trials.		

**Table A12:** Methods of determining *ρ_max_* according to PN-88/B-04481 [64].

Soil Grading	Main Equipment	Procedures to Obtain the Densest Particle Arrangement (Mass and Volume)
*d*_100_ ≤ 5 mm, *d*_90_ ≤ 2 mm	Mould: *V_m_* = 500 cm^3^, *D_m_* = 70 mm; top plate with a hook, vibration fork (550 g)	After determining *ρ_min_*, the top plate is placed on the soil surface; the material is compacted for 1 min by tapping the vibrating fork against the mould walls; at first, lightly and slowly, then strongly and quickly. Three consecutive measurements of the specimen’s height, each after an additional 30 s. of compaction, should reveal no change.
RESULT: The maximum from 3 trials.		

## Appendix B

Table A13 and Table A14 show the dry densities and optimum water contents of the tested samples. The bold red and green fonts indicate the maximum and minimum dry densities, respectively

**Table A13:** Dry densities [g/cm^3^] and optimum water contents *w_opt_* [%] of the single-material samples.

Sample	at *w* < 1%							at *w* ≈ 5%			at *w_opt_*	
	*ρ_d,min_*	*ρ_d,max_*	*ρ_d,Pr_*	*ρ_d_*_,25kPa_	*ρ_d_*_,50kPa_	*ρ_d_*_,100kPa_	*ρ_d_*_,200kPa_	*ρ_d,min_*	*ρ_d,max_*	*ρ_d,Pr_*	*w_opt_*	*ρ_d,max,Pr_*
C(w)	1.285	1.657	1.710	1.525	1.544	1.556	1.574	**1.282**	1.736	1.710	17.0	**1.784**
C(u)_R_	1.181	1.405	1.474	1.343	1.360	1.363	1.383	**1.159**	1.467	1.470	16.5	**1.644**
S(u)	1.387	**1.704**	-	1.564	1.576	1.615	1.608	**0.865**	1.549	1.550	15.9	1.579
R(u)	0.433	0.542	-	0.638	0.696	0.756	**0.842**	**0.407**	0.562	0.630	16.5	0.612
R(u)_S_	0.309	0.597	0.522	-	0.637	0.690	**0.739**	**0.296**	0.577	0.529	46.0	0.594
R(w)	0.436	0.720	0.726	0.679	0.717	0.763	**0.819**	**0.419**	0.678	0.738	19.3	0.757

**Table A14:** Dry densities [g/cm^3^] and optimum water contents *w_opt_* [%] of the RCA-TDA mixtures.

Sample	at *w* < 1%							at *w* ≈ 5%			at *w_opt_*	
	*ρ_d,min_*	*ρ_d,max_*	*ρ_d,Pr_*	*ρ_d_*_,25kPa_	*ρ_d_*_,50kPa_	*ρ_d_*_,100kPa_	*ρ_d_*_,200kPa_	*ρ_d,min_*	*ρ_d,max_*	*ρ_d,Pr_*	*w_opt_*	*ρ_d,max,Pr_*
CR(w)/11%/>5.6 mm	1.015	1.481	1.506	1.340	1.353	1.353	1.379	**1.000**	1.435	1.508	16.4	**1.548**
CR(w)/11%	1.045	1.473	1.481	1.327	1.353	1.372	1.405	**1.016**	1.493	1.526	14.3	**1.662**
CR(w)/11%/≤0.25 mm	1.121	1.479	1.527	1.390	1.451	1.460	1.510	**1.109**	1.483	1.514	14.7	**1.564**
CR(w)/23%/>5.0 mm	0.813	1.360	1.298	1.162	1.177	1.250	1.245	**0.761**	1.336	1.318	17.0	**1.372**
CR(w)/23%	**0.892**	1.364	1.296	1.230	1.250	1.265	1.304	0.912	1.329	1.308	17.7	**1.392**
CR(w)/55%/>2.0 mm	0.607	**1.006**	0.948	0.921	0.935	1.000	1.039	**0.562**	0.939	0.957	12.0	0.962
CR(w)/55%	0.613	0.970	0.989	0.919	0.958	1.004	**1.066**	**0.601**	1.012	1.009	25.2	1.040

**Table A15:** Dry densities [g/cm^3^] of the sand–TDA mixtures.

Sample	at *w* < 1%						at *w* ≈ 5%		
	*ρ_d,min_*	*ρ_d,max_*	*ρ_d_*_,25kPa_	*ρ_d_*_,50kPa_	*ρ_d_*_,100kPa_	*ρ_d_*_,200kPa_	*ρ_d,min_*	*ρ_d,max_*	*ρ_d,Pr_*
SR(u)/15.4%	1.149	**1.533**	1.255	1.288	1.327	1.338	**0.815**	1.381	1.460
SR(u)/26.6%	1.006	**1.426**	1.142	1.190	1.242	1.279	**0.740**	1.232	1.290
SR(p)/42.1%	0.825	**1.181**	1.009	1.052	1.117	1.161	**0.645**	1.032	1.140
